# Perisomatic Inhibition and Its Relation to Epilepsy and to Synchrony Generation in the Human Neocortex

**DOI:** 10.3390/ijms23010202

**Published:** 2021-12-24

**Authors:** Estilla Zsófia Tóth, Felicia Gyöngyvér Szabó, Ágnes Kandrács, Noémi Orsolya Molnár, Gábor Nagy, Attila G. Bagó, Loránd Erőss, Dániel Fabó, Boglárka Hajnal, Bence Rácz, Lucia Wittner, István Ulbert, Kinga Tóth

**Affiliations:** 1 Research Center for Natural Sciences, Institute of Cognitive Neuroscience and Psychology, Eötvös Loránd Research Network, 1117 Budapest, Hungary; toth.estilla.zsofia@ttk.hu (E.Z.T.); agnes.kandracs@gmail.com (Á.K.); noemiomolnar@gmail.com (N.O.M.); ulbert.istvan@ttk.hu (I.U.); toth.kinga@ttk.hu (K.T.); 2 Szentágothai János Doctoral School, Semmelweis University, 1026 Budapest, Hungary; haboglarka90@hotmail.com; 3 Faculty of Information Technology and Bionics, Péter Pázmány Catholic University, 1083 Budapest, Hungary; szabgyf@gmail.com; 4 National Institute of Mental Health, Neurology and Neurosurgery, 1145 Budapest, Hungary; gnagydr@gmail.com (G.N.); bagoatt@hotmail.com (A.G.B.); l.g.eross@gmail.com (L.E.); fabo.daniel@gmail.com (D.F.); 5 Department of Anatomy and Histology, University of Veterinary Medicine, 1078 Budapest, Hungary; racz.bence@univet.hu

**Keywords:** perisomatic inhibition, basket cell, human, epilepsy, synchrony, electron microscopy

## Abstract

Inhibitory neurons innervating the perisomatic region of cortical excitatory principal cells are known to control the emergence of several physiological and pathological synchronous events, including epileptic interictal spikes. In humans, little is known about their role in synchrony generation, although their changes in epilepsy have been thoroughly investigated. This paper demonstraits how parvalbumin (PV)- and type 1 cannabinoid receptor (CB1R)-positive perisomatic interneurons innervate pyramidal cell bodies, and their role in synchronous population events spontaneously emerging in the human epileptic and non-epileptic neocortex, in vitro. Quantitative electron microscopy showed that the overall, PV+ and CB1R+ somatic inhibitory inputs remained unchanged in focal cortical epilepsy. On the contrary, the size of PV-stained synapses increased, and their number decreased in epileptic samples, in synchrony generating regions. Pharmacology demonstrated—in conjunction with the electron microscopy—that although both perisomatic cell types participate, PV+ cells have stronger influence on the generation of population activity in epileptic samples. The somatic inhibitory input of neocortical pyramidal cells remained almost intact in epilepsy, but the larger and consequently more efficient somatic synapses might account for a higher synchrony in this neuron population. This, together with epileptic hyperexcitability, might make a cortical region predisposed to generate or participate in hypersynchronous events.

## 1. Introduction

Epilepsies are thought to be associated with neuronal hypersynchrony, resulting in the generation and maintenance of paroxysmal activity, such as interictal spikes and seizures. Understanding the role of different neuron types in the generation of synchronies is crucial to identify what makes a brain region predisposed to generate epileptic events. A finely tuned balance of excitatory and inhibitory neuronal activity is required for the emergence of different physiological oscillations and synchronous events [1,2], constituting the neurophysiological basis for cognitive processes. This balance is perturbed in epilepsy, resulting in the emergence of pathological synchronisations [3]. Epileptic reorganization and the role of excitatory and inhibitory cell types in the initiation of synchronous activity has been widely studied in animal models (for reviews see [4,5,6,7,8]), but significantly less is known about human neuronal network (for reviews see [9,10]). In this study, we elucidate the reorganization of inhibitory neurons in the human epileptic neocortex and its relationship to the generation of synchronous events.

Synchronous population activity (SPA) emerges spontaneously in the surgically removed brain tissue of both epileptic and non-epileptic (tumor) patients, in vitro [11,12,13,14]. SPA occurrence is similar in the different lobes of the brain but is more probable in epileptic than in non-epileptic patients [15]. These population events are different from epileptiform interictal spikes and are considered to be non-epileptic synchronies [15,16,17]. SPA seems to be a human-specific synchrony type, since no similar population activity could be detected in the neocortical slice preparations of any other species examined. Recently described human-specific neuron type [18] and connectivity rules [19] might underlie the ability of the human neocortex to generate this special synchrony. Although SPAs are generally similar in epileptic and non-epileptic tissue, the subtle differences point to a higher excitability and synchrony in the epileptic neocortex which might be related to the emergence of paroxysmal activity in these patients [15,16].

Inhibitory interneurons innervating the perisomatic region of principal cells are known to efficiently control the output, while inhibitory cells terminating on the dendritic region of principal cells regulate their input [20]. Being able to modulate the activity of a large group of excitatory cells, perisomatic inhibitory interneurons were shown to play a crucial role both in physiological and pathological network synchronies (for review see [21]). Two non-overlapping populations of perisomatic inhibitory interneurons were identified in different cortical regions, the parvalbumin (PV)-containing basket- and axo-axonic cells [22] and the cholecystokinin (CCK)/type 1 cannabinoid receptor (CB1R)-expressing regular spiking basket cells [21]. Basket cells form synapses on the cell body, whereas axo-axonic or chandelier cells terminate on the axon initial segment [23] of their target neurons. The essential role of the fast-spiking PV-positive basket cells was shown in case of several physiological [24,25,26] and epileptic [26,27,28,29,30] synchronous processes. While PV-positive perisomatic cells are specialized to tightly control rhythmic oscillatory activity, CCK/CB1R-positive neurons are involved in the fine-tuning of synchronous ensemble activities [21]. In addition, the CCK/CB1R-positive basket cell type was also found to participate in several synchronous activities [31,32,33,34], including epileptic interictal spikes [35].

Similar to rodents [36], PV is present in perisomatic basket and axo-axonic cells in the human neocortex [37,38]. PV stains multipolar cells with long and aspiny dendrites crossing several neocortical layers, and axons giving mainly perisomatic synapses or arriving from thalamocortical projections [38]. The reorganization of the PV-positive interneurons has been described in epilepsy. The number of PV-stained neurons was found to decrease in the epileptic human neocortex by several research groups [15,39,40], although both the overall optical density of PV-immunoreactivity and the number of PV-positive cells were shown to be unchanged in the human epileptic middle temporal gyrus by one study [11]. Decrease in the density of inhibitory synapses [40] and the loss of perisomatic inhibitory synapses [41] was demonstrated in abnormal neocortical regions of epileptic patients. Modifications of the PV-positive inhibitory network and the perisomatic inhibition in the epileptic hippocampus seems complex (for review see [42]). Although the number of PV-stained cells decreased in epilepsy, the perisomatic inhibition of the principal cells is preserved is the Cornu Ammonis [43,44] and is increased in the dentate gyrus [45]. The distribution of the target cellular compartments has also changed in a region-dependent manner [42].

CB1R is present both in the monkey and human neocortex, with a different laminar distribution across different brain regions [46]. CB1R-staining labels another, non-overlapping perisomatic inhibitory neuron type, the regular spiking basket cells, also containing the neurochemical marker: cholecystokinin (for review see [21]). CB1R was also found in asymmetric, excitatory terminals in the rodent hippocampus [47], but the mRNA level of the CB1R was 20-fold higher in GABAergic than in excitatory cells [48]. In the monkey neocortex CB1R was only found in terminals giving exclusively symmetric (inhibitory) synapses [46]. The overexpression of the CB1R was demonstrated in the epileptic neocortex related to malformations in cortical development such as dysplasias and glioneural tumors [49]. Changes in the levels of CB1R were more complex in the hippocampus of patients with pharmacoresistant MTLE: there was a reduction in the number of excitatory synapses, an effect associated with a low expression of CB1Rs [50], whereas the inhibitory presynaptic terminals showed an enhanced expression of CB1Rs [51].

Acetylcholine receptors are widely distributed in the brain, and the activation of both ionotropic nicotinic (nAChR) and metabotropic muscarinic receptors (mAChR) is implicated in numerous cognitive functions, such as arousal, learning, and memory. The activation of the nAChRs enhances GABAergic inhibition arriving to both excitatory and inhibitory cells in the hippocampus and the neocortex [52] through a variety of pre- and postsynaptic signals [53]. Acetylcholine—via mAChRs—can modulate the activity of both excitatory pyramidal cells and of perisomatic inhibitory interneurons (for review see [54]). The activation of M1-type mAChRs inhibits layer 5 pyramidal cells in the rodent neocortex [55], which is followed by a long-lasting, voltage-dependent excitation [56,57]. In the case of PV-containing perisomatic inhibitory cells, the activation of M2-type mAChRs located on their axon terminals directly diminishes the GABA release [32,58,59]. Furthermore, in the human visual cortex, about 75% of the PV-positive cells also contain the M1-type receptor [60], which might provide further inhibition of these neurons upon cholinergic stimulation. In case of CB1R-expressing perisomatic inhibitory interneurons, cholinergic activation decreases GABA release via an indirect way: the activation of the postsynaptically located M1/M3 mAChRs on pyramidal cells triggers the synthesis of endocannabinoids [32,61], which leads to the inhibition of the presynaptic neurotransmission [62,63,64] through a retrograde signaling pathway [65,66]. Alteration in mAChR function was shown to play a role in epileptogenesis both in animal models [67,68], as well as in epileptic human neocortical slice preparations [69]. Although carbachol (CCh) is an agonist for both nicotinic and muscarinic receptors, it was found to mainly produce muscarinic effects, in vitro [70,71].

The aim of this study was to describe the alterations in the distribution and the output connections of the two types of perisomatic inhibitory cells in the human neocortex in epilepsy, as well as to elucidate the possible role of these neuron types in the generation of synchronous population activity. We examined the distribution of PV- and CB1R-positive elements in the human epileptic and non-epileptic neocortex and made a quantitative electron microscopic analysis concerning the perisomatic inhibitory input of supragranular pyramidal cell with a focus on PV- and CB1R-positive axon terminals. Furthermore, we investigated the participation of the two perisomatic inhibitory cell types in the emergence of SPA by selectively modulating the cholinergic system.

## 2. Results

### 2.1. Light Microscopy of Perisomatic Inhibitory Cells

#### 2.1.1. Parvalbumin-Positive Interneurons

**Figure 1:**
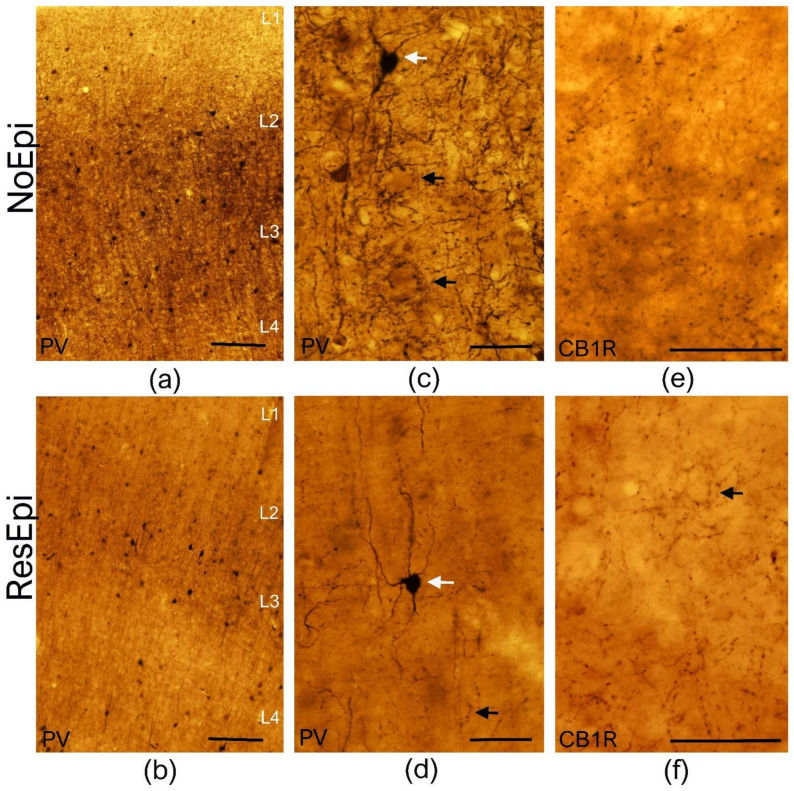
Light microscopy of the human temporal neocortex. Low magnification light microscopic images show the distribution of PV-positive elements in the human temporal neocortex derived from NoEpi (**a**) and ResEpi (**b**) slices. Numerous PV-positive interneurons were found scattered throughout the neocortical layers both in NoEpi and in ResEpi samples. High magnification images (**c**,**d**) show that PV stains multipolar neurons with aspiny dendrites (white arrows on (**c**,**d**)), and a homogeneous axonal meshwork. A dense axonal bundle is visible in layer 3 in the neocortex of both NoEpi (**a**,**c**) and ResEpi (**b**,**d**) patients. Black arrows point to the typical basket formations of PV-stained axons. CB1R is expressed only in axons of perisomatic cells in the non-epileptic (**e**) and epileptic (**f**) human neocortex. The axonal cloud was homogeneous in layer 2/3, and several basket formations were visible (black arrow on (**f**)). Scale bars: (**a**,**b**): 200 µm, (**c**–**f**): 50 µm.

PV is a Ca^2+^-binding protein present in one group of perisomatic inhibitory cells in the human neocortex [37,38]: the fast-spiking basket cells innervating the somata, and the chandelier cells specialized to terminate on the axon initial segment of cortical neurons. We performed PV-immunostaining in neocortical samples derived from both non-epileptic (*n* = 12) and epileptic patients (*n* = 12, for patient data see Table 1). As described previously [37], PV labeled non-pyramidal cells in all layers of the non-epileptic (Figure 1a,c) and epileptic (Figure 1b,d) human neocortex. These cells possessed multipolar cell body (white arrows on Figure 1c,d), long aspiny dendrites, traveling through several layers. PV-positive axon terminals were visible in every neocortical layer, with a considerably denser axonal bundle located in layer 3 (Figure 1a,b). In agreement with our previous work [15], the number of PV-positive cell bodies slightly decreased in the neocortex of ResEpi compared to NoEpi patients. Axons forming the typical basket formations (black arrows on Figure 1c,d) were visible in both patient groups. In some cases, the typical axo-axonic formations were also visible, mainly in the infra-granular layers (not shown, in layer 5 and 6).

#### 2.1.2. CB1 Cannabinoid Receptor-Positive Inhibitory Cells

Type 1 cannabinoid receptor (CB1R) is present in another, non-overlapping perisomatic inhibitory neuron type than PV, the regular spiking basket cells, also containing the neurochemical marker cholecystokinin [21]. In contrast to the hippocampus, where asymmetric, excitatory terminals also contain low levels of CB1R [48], in the neocortex CB1R was only found in cells giving exclusively symmetric (inhibitory) synapses both in rodents [48] and primates [46]. We examined the distribution of CB1R+ elements in the temporal neocortex of human NoEpi (*n* = 9) and ResEpi (*n* = 8) patients, respectively. In the neocortex of NoEpi and ResEpi patients the CB1R-positive terminals showed a similar distribution: a homogeneous axonal cloud was visible in all neocortical layers (Figure 1). As previously described in the monkey brain [46], in the human parietal cortex a denser axonal bundle appeared in layer 4; whereas, in the temporal lobe, a denser bundle was located in layers 1 and 2. In some cases, basket-like formations were also visible, however, these were not as evident as PV-positive baskets (see Figure 1). We could not see considerable changes in the density of the CB1R+ axonal network in the epileptic tissue compared to non-epileptic samples.

### 2.2. Electron Microscopy

**Figure 2:**
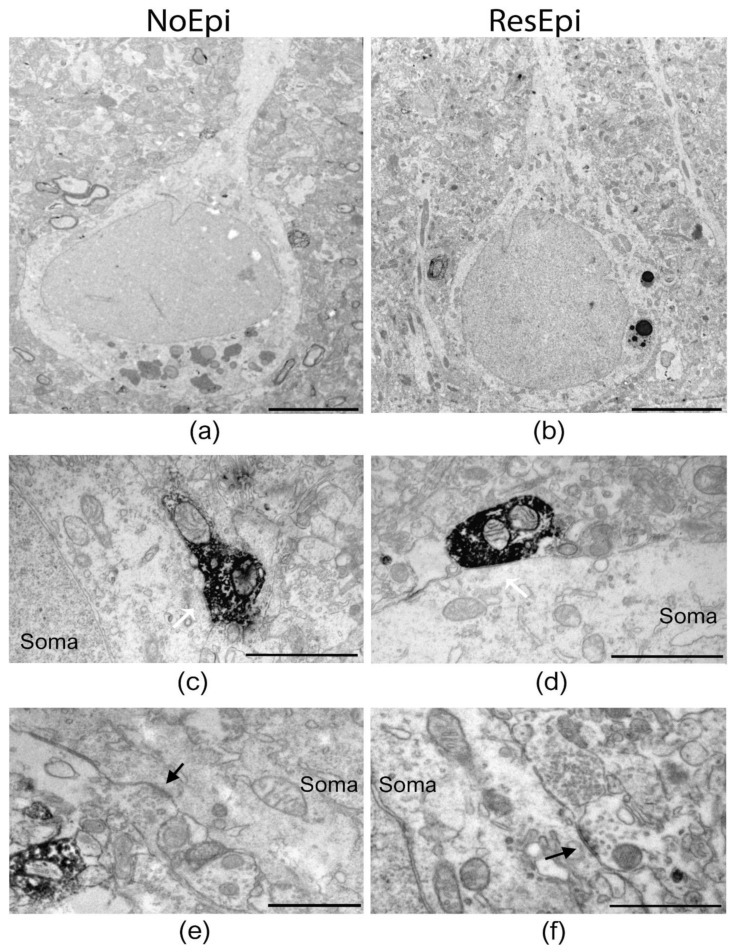
Electron microscopy of temporal neocortical slices stained with PV. Low magnification electron micrographs (**a**,**b**) show layer 2/3 pyramidal cells in the temporal neocortex of NoEpi (**a**) and ResEpi (**b**) patients in sections stained with the perisomatic interneuron marker PV. High magnification electron microscopic images show PV-positive axon terminals giving symmetric (presumably inhibitory) synapses to the soma of layer 2/3 pyramidal cells (white arrows on (**c**,**d**)), both in non-epileptic (**c**) and epileptic (**d**) tissue. Non-stained boutons also terminated on the cell body membrane of the pyramidal cells (black arrow on (**e**,**f**)), both in NoEpi (**e**) and ResEpi (**f**). All pictures were taken from regions where SPA was generated. Scale bars: (**a**,**b**): 5 µm, (**c**–**f**): 1 µm.

**Figure 3:**
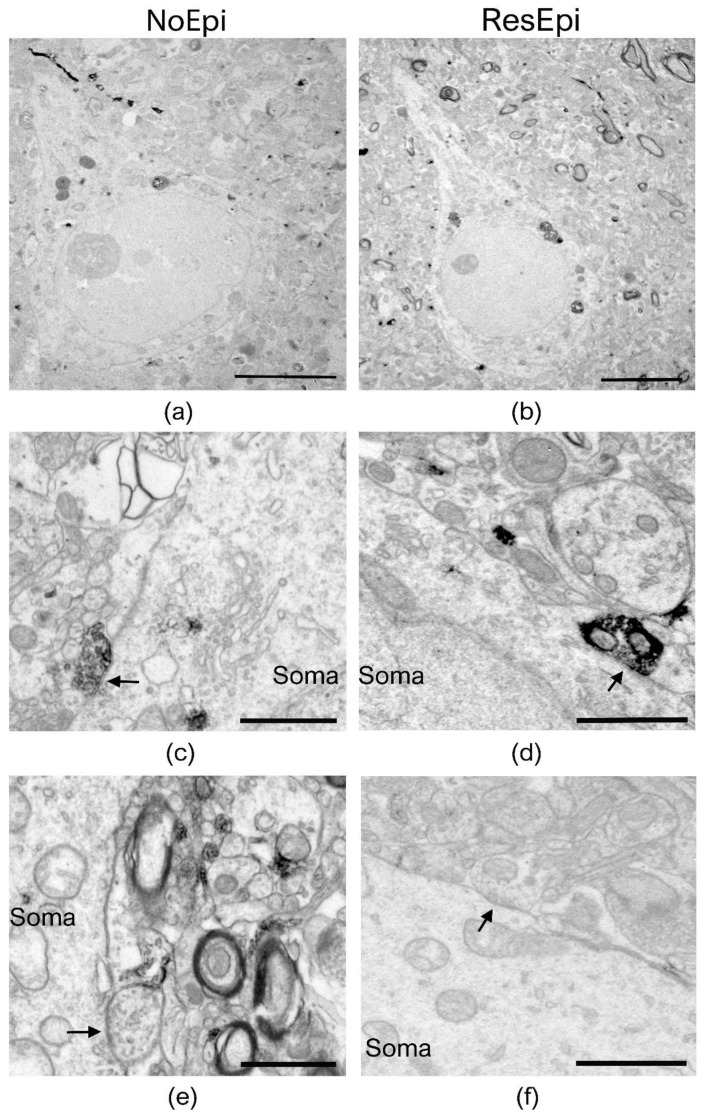
Electron microscopy of temporal neocortical slices stained with CB1R. Layer 2/3 pyramidal cells are shown on low magnification electron micrographs, taken from sections stained with the perisomatic interneuron marker CB1R, derived from non-epileptic (**a**) and epileptic (**b**) patients. Both CB1R+ (**c**,**d**) and CB1R– (**e**,**f**) axon terminals give symmetric (inhibitory) synapses to the cell body (soma) of the pyramidal neurons (black arrows). All pictures were taken in regions where SPA was generated in electrophysiological recordings. Scale bars: (**a**,**b**): 5 µm, (**c**–**f**): 1 µm.

Quantitative electron microscopy was performed to examine the synaptic input of human neocortical pyramidal cells, as well as to describe the distribution of basket cell axon terminals in relation to epilepsy and to the generation of SPA. As SPA was generated most frequently in the supragranular layers (layers 1–3, see later), we examined layer 2/3 pyramidal cells. Since CB1R has a different laminar distribution across different cortical areas, for the electron microscopy we chose only samples derived from the temporal lobe. Sections stained for either PV- or CB1R-positive interneurons were investigated to determine the ratio of the axon terminals belonging to the two types of basket cells both in epileptic and in non-epileptic tissue (Figure 2 and Figure 3). To reveal the possible role of these interneurons in synchrony generation, we studied two regions in each section: one generating and one lacking SPA.

We examined two regions of interest from each slice (one generating and one lacking SPA) and used six PV-stained slices (from three NoEpi and three ResEpi patients) and six CB1R-stained slices (from three NoEpi and three ResEpi patients). Altogether we examined 24 regions of interest from 12 slices. We examined the perisomatic input of 123 cells in four NoEpi patients (13.1 ± 4.8 pyramidal cell somata per region of interest) and 157 pyramidal cells in six ResEpi samples (10.3 ± 3.7 cell bodies per region of interest). We detected altogether 279 and 387 synapses giving contacts to pyramidal cell somata in NoEpi and ResEpi tissue, respectively (Table 2).

#### 2.2.1. Changes Related to Epilepsy

**Figure 4:**
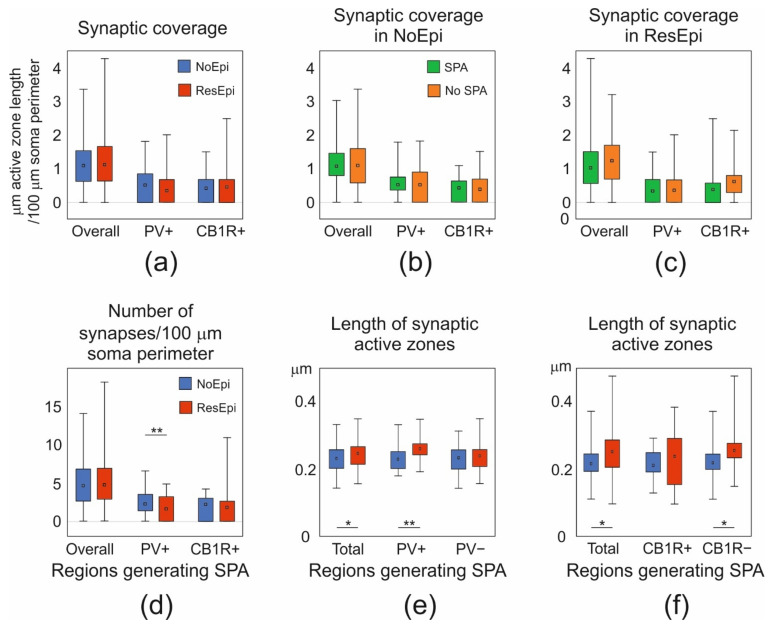
(**a**) The overall synaptic coverage (µm active zone length/100 µm soma perimeter) of layer 2/3 pyramidal cells, and synaptic coverage arriving from PV- or CB1R-positive boutons were all similar in NoEpi (blue) and ResEpi (red) slices (Mann–Whitney U test, *p* > 0.05). (**b**,**c**) The overall, the PV+, and CB1R+ synaptic coverage in regions generating (SPA, green) and lacking (No SPA, orange) SPA was similar both in NoEpi (**b**) and ResEpi (**c**) slices (Mann–Whitney U test, *p* > 0.05). (**d**) The total number of synapses/100 µm soma perimeter within regions generating SPA was similar in NoEpi and ResEpi slices (Mann–Whitney U test, *p* > 0.05), but the number of PV+ synapses/100 µm soma perimeter was significantly lower in ResEpi compared to NoEpi tissue (Mann–Whitney U test, *p* < 0.01). The number of CB1R+ synapses/100 µm soma perimeter did not change in epilepsy (Mann–Whitney U test, *p* > 0.05). (**e**) The length of the synaptic active zones in cortical regions generating SPA was significantly higher in ResEpi than in NoEpi slices, which difference came from PV+ boutons (Student’s *t*-test, *p* < 0.001). The active zone length of PV-negative terminals was similar in ResEpi and NoEpi tissue. (**f**) The increase in the active zone length of PV+ boutons was also detectable in CB1R-stained sections: the average active zone length of all and of CB1R-negative boutons were higher in ResEpi than in NoEpi tissue (Student’s *t*-test, *p* < 0.05). All values are shown in median (first–third quarters), * *p* < 0.05, ** *p* < 0.01.

Inhibitory interneurons were shown to participate in the epileptic reorganization in the human epileptic hippocampus [72] and neocortex [40]. We aimed to get insights into the mechanisms of synaptic reorganisation in the human epileptic neocortex affecting perisomatic inhibition. Therefore, we examined the distribution of synaptic contacts terminating on pyramidal cells in NoEpi vs. ResEpi slices, irrespective of the emergence of SPA. We found only symmetrical synapses (presumably inhibitory) on the cell body membrane of the examined cells (Figure 2 and Figure 3). We calculated the synaptic coverage of each neuron—i.e., µm synaptic active zone/100 µm soma perimeter. The overall synaptic coverage of layer 2/3 pyramidal cells did not change in epilepsy (1.12 [0.64–1.67] µm synaptic active zone/100 µm soma perimeter in ResEpi) compared to NoEpi samples (1.07 [0.62–1.50] µm synapse/100 µm soma perimeter, *p* > 0.05, Mann–Whitney U test, Table 2, Figure 4). As most of our data did not follow normal distribution, we provide median [first to third quartiles] in most cases.

The size of the synaptic terminals was found to be larger in the human epileptic hippocampus, compared to non-epileptic tissue [73]. To see whether similar processes occur in the neocortex, we assessed the average length of the synaptic active zones of the boutons giving synapse to the pyramidal cell bodies. We did not find differences between NoEpi and ResEpi samples, neither between regions generating and lacking SPA (Kruskal–Wallis ANOVA, *p* > 0.05, for exact values see Table 2).

#### 2.2.2. Changes in the Two Perisomatic Basket Cell Axonal Clouds

The synaptic reorganization of the PV-positive interneurons has been thoroughly described in the epileptic human hippocampus (for review see [42]). Depending on the region, the perisomatic inhibition of the excitatory principal cells showed different changes. To assess the possible epileptic reorganization of the two types of basket cells in epilepsy, we determined the ratio of PV- or CB1R-positive boutons among all terminals giving synapses to pyramidal cell somata and the PV+ or CB1R+ synaptic coverage of pyramidal cells, both in NoEpi and ResEpi samples.

The ratio of PV-stained boutons was 46.75 [0.00–58.41] % in NoEpi samples, and was not different in ResEpi slices, 30.18 [0.00–54.44] %. The proportion of CB1R-positive axon terminals was 42.82 [0.22–57.28] % in NoEpi, and 44.66 [22.23–62.72] % in ResEpi tissue (none are significantly different, Chi-square test *p* > 0.05). The synaptic coverage arriving from PV- or CB1R-positive boutons was similar in NoEpi and ResEpi samples, and in regions generating and lacking SPA (Kruskal–Wallis ANOVA, *p* > 0.05, for exact values see Table 2).

#### 2.2.3. Differences between Regions Generating and Lacking SPA

In our previous study, we showed that both neocortical pyramidal cells and inter-neurons are involved in the generation of SPA [16]. Differences in the perisomatic inhibition of pyramidal cells could account for a higher excitability or a higher synchrony of the excitatory neuronal population (for reviews see [21,42,74]), and thus, could be related to the generation of SPA. To test this hypothesis, we compared the synaptic coverage of pyramidal cell bodies located in regions generating and lacking SPA. We examined NoEpi and ResEpi samples separately and found no significant differences in the overall synaptic coverage between regions initiating SPA (NoEpi: 1.07 [0.80–1.46], ResEpi: 1.03 [0.56–1.51]) and lacking SPA (NoEpi: 1.07 [0.55–1.52], ResEpi: 1.23 [0.69–1.70], Kruskal–Wallis ANOVA on Ranks, *p* > 0.05, Table 2, Figure 4).

Further analyses revealed that in regions generating SPA, the active zone length of PV+ terminals were higher in ResEpi compared to NoEpi samples (NoEpi: 0.23 ± 0.04, 0.23 [0.20–0.25], ResEpi: 0.26 ± 0.04, 0.26 [0.24–0.27], Student’s *t*-test *p* = 0.01, Table 2, Figure 4). In line with this, the non-stained axon terminals of CB1R-immunostained sections possessed longer active zones in ResEpi than in NoEpi slices (NoEpi: 0.22 ± 0.05, 0.22 [0.20–0.25], ResEpi: 0.25 ± 0.05, 0.25 [0.23–0.28], Student’s *t*-test *p* = 0.05, Table 2, Figure 4), only in regions where SPA emerged. Since the PV-positive synaptic coverage of the pyramidal cell bodies was similar in NoEpi and ResEpi tissue, this result implies that the number of PV+ boutons might be lower in epileptic slices. Indeed, we found a statistical difference in the number of PV+ terminals/100 µm soma perimeter between NoEpi and ResEpi slices (NoEpi: 2.23 [1.36–3.51], ResEpi: 0.23 [0.00–1.90], Mann–Whitney U test *p* = 0.01, Table 2, Figure 4), in regions generating SPA.

### 2.3. Role of Perisomatic Inhibitory Cells in Synchrony Generation

**Figure 5:**
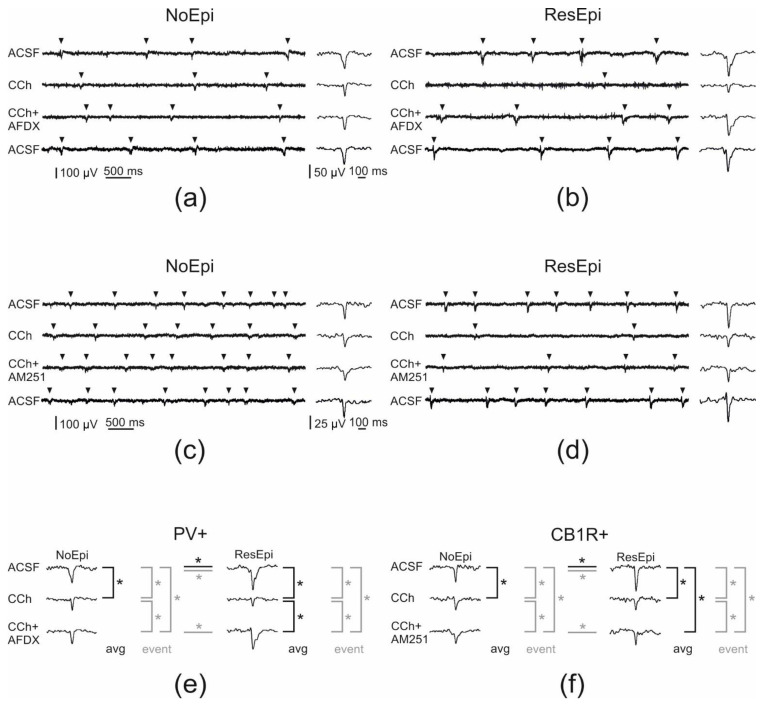
Pharmacological modifications of SPAs generated in the human non-epileptic and epileptic neocortex. Arrowheads point on SPA events on (**a**–**d**). In NoEpi slices carbachol (CCh) had no effect on the recurrence frequency of SPA, though it reduced the amplitude of the LFPg compared to the baseline (ACSF, (**a**,**c**)). In ResEpi tissue, CCh induced a considerably stronger effect on SPA. It reduced the recurrence frequency and the LFPg amplitude to lower levels in ResEpi than in NoEpi cases (**b**,**d**). Applying AF-DX 116—the M2 mAChR antagonist—together with CCh had no effect on the frequency of events in the NoEpi cases. However, it partially restored the LFPg amplitude (**a**). In ResEpi slices, AF-DX recovered the frequency of the events and significantly increased the amplitude of the LFPg compared to that of CCh (**b**). In NoEpi cases, the frequency of the events was comparable with the baseline when applying AM251—the CB1R antagonist—together with CCh. LFPg amplitude was higher during co-application than during CCh alone, but still lower than in physiological solution (**c**). In ResEpi slices, both the frequency of the events and the LFPg amplitude showed a small recovery when we applied CCh and AM251 together (**d**). The effect of all three pharmacological agents could be washed out, the frequency and LFPg amplitudes returned to the levels of the baseline (lowest sweeps, ACSF). (**e**,**f**) show the significant differences (labeled with asterisk) we found in the LFPg amplitude during the application of AF-DX 116 (**e**) and AM251 (**f**). We compared the averaged LFPg amplitudes (avg, black) and the LFPg amplitudes of all individual SPA events (event, grey) with one-way repeated measures ANOVA. Black lines represent significant differences in the averaged LFPg amplitudes, grey colours depict significant differences when analysing the LFPg amplitude of individual SPA events. Differences found between NoEpi and ResEpi cases (Welch’s *t*-test) are labelled with horizontal lines.

Spontaneous synchronous population activity emerged in neocortical slices derived from both epileptic and non-epileptic patients, in physiological bath solution (see also [12,13,15,17]). Pharmacological and clustering data in earlier work [15,16] suggested that inhibitory cells participate in the generation of SPA. In this study, we examined SPA emerged in 12 slices derived from 8 NoEpi patients, and 8 slices from 6 ResEpi patients, and focused on the role of perisomatic inhibitory cells. Note that SPA designates the totality of recurring synchronous events in a given recording. SPA emerged in physiological bath solution in both epileptic and non-epileptic tissue. It was detected in most cases in the supragranular layers (in layers 1–3): in 11/12 slices in NoEpi, and in 6/8 slices in ResEpi slices. The remaining SPAs were generated in the granular-infragranular layers (layers 4–6). The recurrence frequency of the SPA was 1.37 [0.74–1.76] Hz in NoEpi and 0.88 [0.67–1.84] Hz in ResEpi slices (not different, Welch’s *t*-test, *p* > 0.05, Table 3). The LFPg amplitude was 36.30 [25.01–46.44] µV in NoEpi, and in agreement with our previous studies [15,16], it was significantly higher in ResEpi tissue: 66.01 [36.46–106.49] µV (Welch’s *t*-test, *p* = 0.035, Table 4, Figure 5). The multiple unit activity (MUA) amplitude was 0.67 [0.56–1.10] µV and 3.12 [1.82–5.36] µV in NoEpi and ResEpi slices, respectively (significantly different, Welch’s *t*-test, *p* = 0.035, Table 5).

**Figure 6:**
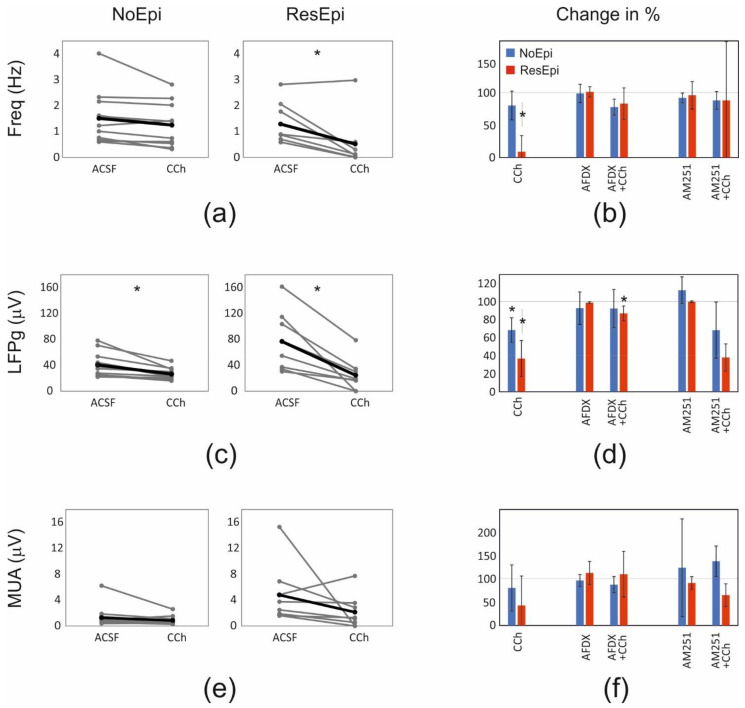
Quantifications of the pharmacological changes. Analyses using the frequency, LFPg and MUA averages are shown. Graphs showing the changes in the recurrence frequency (**a**,**b**), LFPg (**c**,**d**) and the MUA (**e**,**f**) amplitudes of SPAs spontaneously generated by NoEpi and ResEpi slices, during the application of the CCh (**a**,**c**,**e**) and the antagonists preventing the effect of CCh on PV+ (AFDX) and CB1R+ (AM251) neurons (**b**,**d**,**f**). In NoEpi slices CCh reduced the LFPg amplitude (**c**), but not the frequency (**a**) or the MUA amplitude (**e**). In ResEpi slices CCh exerted a larger effect, reducing both the recurrence frequency and the LFPg amplitude (**a**,**c**), but not the MUA (**e**). Grey lines designate single experiments, black lines show the average. The changes in the frequency (**b**), LFPg (**d**) and MUA (**f**) amplitudes, compared to the baseline period (ACSF) are shown in percentage. 100% represents the value of the baseline period preceding the drug application. Asterisks show significant differences between the values obtained during the baseline and the application of the given pharmacological agent(s). The frequency (**a**,**b**) and the LFPg amplitude (**c**,**d**) change in response to CCh was significantly different in ResEpi compared to the baseline. The LFPg amplitude during CCh application was also significantly lower than in ACSF, in NoEpi slices (**c**,**d**). Preventing the effects of CCh on PV+ neurons by applying AF-DX 116 significantly elevated the LFPg amplitude of SPAs in ResEpi slices compared to the application of CCh (AFDX+CCh on (**d**)). The application of AM-251, which restores the activity of CB1R+ cells, could not exert any significant effect on SPA properties when analysing the averaged LFPg amplitudes. Values on figure (**b**,**d**,**f**) are shown in median ± IQR. * One-way repeated measures ANOVA, *p* < 0.05. Asterisks show statistical significances referring to the differences found with the averaged values. On figure (**b**,**d**,**f**) asterisks show the statistical significances in the effect of the drug application, compared to the baseline (grey line at 100%).

To decrease the GABA release of perisomatic inhibitory cells, carbachol (CCh, 5 µM) was added to the bath solution. Before all drug application a baseline was recorded (spontaneous SPA in physiological solution), and all drug effects were compared to this baseline. Frequency and LFPg values of the baseline were considered to be 100%, when determining drug effects. All pharmacological agents were washed out (also with physiological solution). We have to note that mAChR activation also increases pyramidal cell excitability and firing [75,76], and reduces the amplitude of excitatory synaptic potentials [71], thus, carbachol exerts a mixed effect on both excitatory and inhibitory circuits. Carbachol slightly reduced the recurrence frequency of SPAs in NoEpi slices, to 1.24 [0.59–1.57] Hz (80 [72–95] % compared to the baseline, not significant, one-way repeated measures ANOVA, *p* > 0.05, Table 3, Figure 5 and Figure 6). This effect was considerably more pronounced in ResEpi: in 2/8 cases the SPA disappeared, and in 3/8 cases the recurrence frequency dropped below 30% of the baseline frequency (Figure 5 and Figure 6). On average, the recurrence frequency was 0.10 [0.02–0.37] Hz (9 [3–27] %, significantly lower compared to baseline period, one-way repeated measures ANOVA, *p* = 0.006) in ResEpi slices treated with CCh (Table 3). Regarding the averaged LFPg amplitude, in NoEpi slices it decreased from 36.30 [25.01–46.44] µV to 23.42 [20.00–31.01] µV (68 [63–77] %, significantly lower than during the baseline period, one-way repeated measures ANOVA, *p* = 0.002), whereas the change was more pronounced in ResEpi, it decreased from 66.01 [36.46–106.49] µV to 18.10 [12.20–31.21] µV (37 [25–45] %, significantly lower than baseline, one-way repeated measures ANOVA, *p* = 0.004, but not significantly different from NoEpi, Welch’s *t*-test, *p* > 0.05, Table 4, Figure 5 and Figure 6). Similar results were seen in the LFPg amplitudes, when examining individual SPA events (see Methods/Data Analysis), with much lower *p* values (one-way repeated measures ANOVA, *p* <0.0001 both in NoEpi and ResEpi slices, Table 4). The amplitude of MUA also showed a decrease such as the recurrence frequency and the LFPg amplitude, both in NoEpi (0.67 [0.46–0.85] µV, 81 [57–107] %), and in ResEpi slices (1.21 [0.41–3.03] µV, 43 [22–85] %), however, these differences were not significant compared to the baseline (one-way repeated measures ANOVA, *p* > 0.05, Table 5, Figure 6). The effect of carbachol could be washed out in all cases, the recurrence frequency, the LFPg and MUA amplitudes returned to the levels of the baseline (Figure 5).

#### 2.3.1. Role of PV-Positive Cells

PV-positive interneurons selectively express M2 type mAChRs on their axon ter-minals [77,78]. Blocking these receptors by the selective antagonist AF-DX 116 should prevent the effect of carbachol on this cell type [61]. We added AF-DX 116 (10 µM) + CCh (5 µM) to six NoEpi slices, and the recurrence frequency of SPAs slightly decreased compared to the baseline (one-way repeated measures ANOVA, *p* > 0.05), it became similar to values when CCh was applied alone: 0.82 [0.72–1.19] Hz (78 [75–87] %, Table 3, Figure 5 and Figure 6). In case of ResEpi slices (*n* = 4), AF-DX 116 could largely prevent the effects of CCh: the recurrence frequency changed to 0.90 [0.87–1.07] Hz, (83 [71–96] %), which is comparable with the baseline (one-way repeated measures ANOVA, *p* > 0.05, Table 3, Figure 5 and Figure 6).

Blocking M2 mAChRs had an effect on the LFPg amplitude of SPAs: CCh could reduce the averaged LFPg amplitude in the presence of AF-DX 116 less than alone: to 34.28 [28.52–39.61] µV (92 [75–96] %) in NoEpi and to 63.87 [53.55–76.51] µV (87 [83–91] %) in ResEpi tissue, although these changes were found to be significant only for ResEpi slices (*p* = 0.028, one-way repeated measures ANOVA, Table 4, Figure 5 and Figure 6). To gain more insights, we analysed individual SPA events and found that the LFPg amplitude during co-application with AF-DX 116 was significantly higher than during CCh alone even in NoEpi slices (*p* < 0.0001 both in NoEpi and ResEpi cases), though it was still significantly lower than during the baseline (*p* < 0.0001 both in NoEpi and ResEpi cases, one-way repeated measures ANOVA, Table 4).

MUA amplitude did not change significantly when applying AF-DX 116 and CCh. On average, it became 0.75 [0.69–0.95] µV (88 [80–97] %) in NoEpi and 4.93 [4.21–5.31] µV (111 [86–135] %) in ResEpi slices (one-way repeated measures ANOVA, *p* > 0.05, Table 5, Figure 6). The application of AF-DX 116 without CCh (NoEpi: *n* = 3, ResEpi: *n* = 3 slices, for values see Table 3, Table 4 and Table 5) did not change the recurrence frequency, the LFPg or the MUA amplitudes of SPAs (one-way repeated measures ANOVA, *p* > 0.05, Figure 6).

In summary, blocking the effects of CCh on PV-positive cells partly restored the SPAs. This effect was low in NoEpi, since CCh modifies these SPA parameters at a significantly lower level than in ResEpi samples. Blocking M2 receptors had a more considerable effect in ResEpi tissue: the reduction in the recurrence frequency and LFPg amplitude to CCh were less pronounced, than during the application of CCh alone.

#### 2.3.2. Role of CB1R-Positive Cells

Carbachol decreases the GABA release of CB1R-positive cells through an indirect way. It activates M1/M3 mAChRs on pyramidal cells, which triggers the release of endocannabinoids, acting retrogradely on the CB1Rs [32,61]. To reveal the role of CB1-positive perisomatic inhibitory cells on the generation of SPA, we applied the selective CB1R antagonist AM-251 (1 µM) together with CCh (5 µM). In NoEpi slices the recurrence frequency remained similar to that observed in physiological solution (1.47 [1.03–1.50] Hz, 88 [84–97] %, *n* = 5 slices, Table 3, Figure 5 and Figure 6). In slices derived from ResEpi patients (*n* = 5 slices) AM-251 could not induce significant changes to restore the effects of CCh: the recurrence frequency was 0.83 [0.02–0.94] Hz (88 [2–93] % of the baseline period, not significantly different from CCh, one-way repeated measures ANOVA, *p* > 0.05, Table 3, Figure 5 and Figure 6).

In a bath containing AM251+CCh, the averaged LFPg amplitude slightly decreased both in NoEpi (22.81 [22.34–34.12] µV, 68 [64–95] %) and ResEpi tissue (22.78 [11.18–33.91] µV, 38 [34–50] %), compared to physiological solution. These values were not significantly different from those when only CCh was applied (see earlier, Table 4, Figure 5 and Figure 6, one-way repeated measures ANOVA, *p* > 0.05). When analysing LFPg amplitude of individual SPA events, we found it to be significantly higher during the co-application of AM251 and CCh than during CCh alone, in both patient groups (*p* < 0.0001 both in NoEpi and ResEpi cases). However, both values were still significantly lower than during the baseline (*p* < 0.0001 both in NoEpi and ResEpi, one-way repeated measures ANOVA, Table 4).

MUA amplitude did not change significantly when applying AM251 and CCh (Table 5, Figure 6). On average, it became 1.32 [1.27–1.49] µV (139 [122–155] %) in NoEpi and 2.50 [1.50–4.04] µV (65 [64–88] %) in ResEpi slices (one-way repeated measures ANOVA, *p* > 0.05). The application of AM-251 alone did not cause any change in the recurrence frequency and in the LFPg or MUA amplitudes of the SPAs compared to the baseline (NoEpi: *n* = 3, ResEpi: *n* = 5 slices, Table 3, Table 4 and Table 5, one-way repeated measures ANOVA, *p* > 0.05, Figure 6).

In summary, preventing the blockade of GABA release in CB1R+ cells by applying AM251 could not elicit significant changes in the recurrence frequency of SPA, although a trend of recovery from CCh-induced reduction was observed in ResEpi cases. LFPg amplitude was partially restored both in NoEpi and ResEpi tissues. Note that the magnitude of the recovery achieved by the antagonists in terms of both frequency and LFPg amplitude was much lower in case of AM251, than in case of AF-DX 116 (Table 3 and Table 4, Figure 6).

## 3. Discussion

### 3.1. Changes in the Perisomatic Inhibition Related to Epilepsy

In physiological solution, both the LFPg and MUA amplitudes of SPAs were significantly larger in epileptic compared to non-epileptic tissue (see also [15]). The application of the muscarinic agonist CCh induced the decrease in LFPg amplitude in both patient groups, however, in ResEpi tissue this decrease was more considerate. Furthermore, in ResEpi slices the recurrence frequency was also largely reduced. When studying the role of the two perisomatic inhibitory cell types, we showed that preventing the effects of CCh on PV+ neurons caused a stronger effect in restoring the LFPg decrease in ResEpi than in NoEpi tissue. The modulation of the CB1R+ perisomatic neuron population caused similar effects in epileptic and non-epileptic samples. The increase in the levels of M2 receptors on PV+ cells might result in the more pronounced effect of CCh in epileptic tissue [79]. On the other hand, the higher LFPg and MUA amplitudes in ResEpi suggest that more cells participate in the generation of SPA, which might be also linked the more profound effects of CCh. The epileptic hyperexcitability could account for the involvement of a larger neuron population in the SPA. The loss of inhibition [37,80,81]—especially perisomatic inhibition [82,83]—used to be a tempting theory to explain epileptic hyperexcitability in epilepsy, but was contradicted by a high number of studies (e.g., [42,84]). Our present study also shows that the perisomatic inhibitory input of layer 2/3 pyramidal cells was preserved, despite the decrease of PV-stained neuron numbers [15]. The overall synaptic coverage, the ratio of PV+ and CB1R+ boutons and the synaptic coverage arriving from PV- or CB1R-positive perisomatic interneurons were all similar when comparing NoEpi and ResEpi slices. The only difference we observed in our electron microscopic study was that PV+ boutons were larger and decreased in number in epileptic tissue. These results suggest that—like axons of dendritic inhibitory cells in the neocortical layer 2/3 [15]—the majority of the perisomatic basket cell synapses were not considerably modified in the neocortex of epileptic patients. This contradicts a previous study where the loss of inhibitory synapses was found on the soma and the axon initial segment of pyramidal cells in the peritumoral neocortex of an epileptic patient [41]. For our electron microscopic analysis, we used five epilepsy patients without tumor and one, who had a glioneural tumor located at a distance of >5 cm from the resected neocortical tissue. Thus, the etiology of the patients and the distance from the tumor might account for the differences found in the perisomatic inhibitory input.

The preservation of the overall perisomatic inhibitory input and the ratio of PV+ inhibitory input of neocortical pyramidal cells in epilepsy contrasts to the hippocampal dentate gyrus of temporal lobe epileptic (TLE) patients, where the perisomatic inhibitory input of dentate granule cells has notably increased together with the enhancement of the CB1R+ inhibitory boutons [51] and with the reduction of PV+ cell numbers and ratio of PV+ somatic synapses [45]. The situation in the neocortex is somewhat comparable to hippocampal CA1 and CA2 regions, where the perisomatic synaptic coverage of pyramidal cells and the ratio of PV+ boutons contacting them are preserved in epilepsy as long as the pyramidal cells survive, despite the decreased number of PV+ cell bodies [43,44]. The CB1R+ basket cells can be observed in the epileptic hippocampus, even in the sclerotic CA1 region [50], with an increased density of CB1R+ boutons [42]. Hippocampal granule cells, CA1 and CA2 pyramidal neurons and neocortical (layer 2/3) pyramidal cells belong to different neuron groups, defined by their different gene expression pattern [85,86], neurochemical identity, and their synaptic input–output features [23]. However, neocortical layer 2/3 pyramidal cells show more similarities to hippocampal pyramidal cells than to granule cells in their morphology and physiology [87], such as in their perisomatic inhibitory changes in epilepsy as well. Different cell types might react to, or participate in, the epileptic reorganization with different modifications [88].

Altogether, more profound changes occur in the epileptic hippocampus considering perisomatic inhibition, than what we have observed in the epileptic neocortex [88]. Other reasons besides differences in neuron subtypes might also explain this inconsistency. In pharmacoresistant TLE patients, the hippocampus is usually the region where seizures originate, and not the surrounding temporal neocortex (which was examined in this study). Indeed, all samples included in our electron microscopic study derived from outside of the seizure focus but located in a region recruited during seizure propagation. More severe epileptic reorganization might be associated with a seizure onset zone, than with a surrounding area, and this is the most likely reason why more signs of epileptic reorganization are detectable in the hippocampus, than in the neocortex.

### 3.2. Cholinergic Input of Pyramidal Cells and Epilepsy

Modulation of the muscarinic receptors modifies the behaviour of excitatory cells, not only that of perisomatic inhibitory interneurons. In the healthy brain, the activation of M1 type mAChRs transiently inhibits neocortical pyramidal cells [55], which is followed by a long-lasting, voltage-dependent excitation [56]. If pyramidal cells are depolarized and show continuous firing, the inhibitory action of mAChR activation is more robust and reliable than at resting membrane potential [56]. The neuronal hyperexcitability during epilepsy [15,81] might result in a sustained depolarization of a high number of cells, and higher numbers of neurons firing during SPA. Thus, this voltage-dependent effect can potentially explain why CCh could decrease the activity of more cells in epileptic tissue, and so, could reduce the originally higher LFPg amplitude to the same level, while decreasing the recurrence frequency to a substantially lower level than in non-epileptic tissue.

### 3.3. Perisomatic Inhibitory Interneurons and Synchrony Generation

The knowledge about the initiation mechanism of neuronal synchronies is crucial to understand the possible differences between physiological and pathological synchronies. Perisomatic interneurons—as the most potent cells to inhibit large neuron populations—are indeed efficient in shaping synchronous activities such as sharp-wave ripples [89,90] and interictal-like discharges [35] in the hippocampus. Inhibitory cells were shown to fire at the start of spontaneous [13] and pharmacologically induced [16] epileptiform events in the human neocortex as well. Thus, it is an appealing hypothesis that perisomatic inhibitory cells control the generation of other, non-epileptiform synchronous activities as well, such as SPA.

In the present study, we examined this hypothesis with two different approaches: with pharmacological modulation and quantitative electron microscopic analysis of perisomatic inhibition of neocortical pyramidal cells. Our results showed that perisomatic inhibitory cells indeed contribute to the generation of SPA. By comparing the LFPg amplitudes of the SPA events, we found that modulating the activity of both perisomatic interneuron types partly restored the effect of CCh, both in NoEpi and ResEpi samples (LFPg amplitudes in ACSF > in CCh + antagonist > in CCh). However, when comparing the averages, preventing the effect of CCh on PV+ cells could significantly elevate LFPg amplitude only in ResEpi but not in NoEpi tissue. This latter finding tightly fits to the results of the electron microscopic analysis: only in ResEpi samples, in regions generating SPA, axons of the PV+ (but not the CB1R+ or non-stained) basket cells have been significantly changed. The size of somatic PV+ boutons has significantly increased, together with the decrease in the number of PV+ synapses arriving to the pyramidal cell bodies. The increase in the active zone size is associated to a higher neurotransmitter release, and thus, to a more powerful synaptic transmission [91]. The more efficient PV+ basket cell synapses—although decreased in number—might provide a higher synchrony in the neocortical neuronal network in epilepsy, which can make a region predisposed to generate or participate in hypersynchronous events.

Our pharmacological and electron microscopic results suggest that perisomatic inhibitory cells contribute to the generation of SPAs but do not have an exclusive leading role. This supports our previous studies, where we showed that both excitatory and inhibitory cells are needed for the generation and they both participate in the initiation of SPAs [15,16]. The more pronounced effect of CCh in epileptic tissue, probably assigned to the modulation of pyramidal cell activity, suggests that excitatory cell types also have an important role in the initiation of SPA [16]. We propose that this form of synchrony results from the complex interplay between excitatory and inhibitory neurons, including perisomatic inhibitory cells.

## 4. Materials and Methods

### 4.1. Patients

The patients were operated in the National Institute of Mental Hygiene, Neurology and Neurosurgery (1145 Budapest, Hungary). We received written consent from all patients. Our protocol was approved by the Hungarian Ministry of Health and by the Regional and Institutional Committee of Science and Research Ethics of Scientific Council of Health (ETT TUKEB 20680-4/2012/EKU) and performed in accordance with the Declaration of Helsinki.

### 4.2. Epileptic Patients

Neocortical tissue samples were resected from 12 epileptic patients (Table 1). We obtained tissue from frontal (*n* = 1 patient) and temporal (*n* = 11 patients) lobes. All patients suffered from pharmacoresistant epilepsy (ResEpi, resistant epilepsy) for 22 ± 15 years on average. Epileptic patients were diagnosed with several different pathologies, such as focal cortical dysplasia, cortical gliosis, tumors of glial origin, hippocampal sclerosis, and stroke induced lesion. In the cases of four patients, the obtained tissue was in the seizure onset zone, whereas in the remaining eight patients the seizures invaded that region, but the seizure focus was elsewhere (for details see Table 1). Histopathological changes (signs of dysgenesis or tumor infiltration, Table 1) of the obtained tissue have been verified with Nissl staining, the neuronal marker NeuN-, the astroglial marker glial fibrillary acidic protein- and the two interneuron markers parvalbumin (PV)- or type 1 cannabinoid receptor (CB1R)-immunostainings. Epileptic patients: 5 females, 7 males, age range: 28–53 years, mean ± st.dev.: 37.8 ± 7.7 years.

### 4.3. Non-Epileptic Patients

In this study, we examined 13 patients diagnosed with brain tumor but without epilepsy (NoEpi, no epilepsy, Table 1). These patients—as it is stated in their anamnesis—did not show clinical manifestation of epileptic seizure before the date of their brain surgery. Neocortical tissue was resected from tumor patients from frontal (*n* = 4 patients), temporal (*n* = 5 patients), parietal (*n* = 3 patients), and occipital (*n* = 1 patient) lobes. Thirteen patients were diagnosed with tumors of glial (*n* = 8) or other origins (*n* = 5, for details see Table 1). The resected tissue was always outside of the tumor area. Brain samples deriving from the tumor tissue were excluded from the study. The distance of the obtained neocortical tissue from the tumor (see Table 1) had been assessed by the neurosurgeon, based on magnetic resonance (MR) images, intraoperative pictures and occasionally defined by a navigational system. In case the obtained tissue was closer than 3 cm from the tumor, we considered it ‘close’; if the distance was more than 3 cm, we considered as ‘distant’ tissue. Non-epileptic patients: 9 females, 4 males, age range: 32–81 years, mean ± st.dev.: 60.5 ± 14.2 years.

### 4.4. Tissue Preparation

Tissue was transported from the operating room to the laboratory (located in the same building) in a cold, oxygenated solution containing (in mM) 248 D-sucrose, 26 NaHCO_3_, 1 KCl, 1 CaCl_2_, 10 MgCl_2_, 10 D-glucose and 1 phenol red (Sigma-Aldrich, St. Louis, MO, USA), equilibrated with 5% CO_2_ in 95% O_2_ (Linde Plc, Budapest, Hungary). Neocortical slices of 500 µm thickness were cut with a Leica VT1000S vibratome (Leica Biosystems, Buffalo Grove, IL, USA, RRID:SCR_016495). They were then transferred and maintained at 35–37 °C in an interface chamber, perfused with a standard physiological solution containing (in mM) 124 NaCl, 26 NaHCO_3_, 3.5 KCl, 1 MgCl_2_, 1 CaCl_2_, and 10 D-glucose, equilibrated with 5% CO_2_ in 95% O_2_ (Linde Plc, Budapest, Hungary). If not mentioned separately, Molar Chemicals Ltd. (Halásztelek, Hungary) was the manufacturer of the chemicals.

### 4.5. Recordings

The extracellular local field potential gradient (LFPg) recording was obtained as described previously [14]. Briefly, we used a 24 contact (distance between contacts: 150 µm) laminar microelectrode [42,71], and a custom-made voltage gradient amplifier of pass-band 0.01 Hz to 10 kHz. Signals were digitized with a 32-channel, 16-bit resolution analogue-to-digital converter (National Instruments, Austin, TX, USA) at 20 kHz sampling rate, recorded with a home written routine in LabView8.6 (National Instruments, Austin TX, USA, RRID:SCR_014325). The linear 24 channel microelectrode (Plexon Inc., Dallas, TX, USA) was placed perpendicular to the pial surface, to record from every layer of the neocortex. Slices were mapped from one end to the other at every 300–400 µm.

The presence and the exact location of the SPA was noted in case of every slice. If a stable SPA was observed (persisting without evident changes for more than 10 min), pharmacological experiment was performed. The non-selective acetyl choline receptor (AChR) agonist carbachol (CCh) was applied first (5 or 10 µM, 100 mL, approximately 20–30 min, obtained from Merck, Kenilworth, NJ, USA) to decrease the GABA release from the perisomatic inhibitory cells [32]. After a first washout period (100 µL solution or 30 min), either the M2 muscarinic acetyl choline receptor (mAChR) antagonist AF-DX 116 (10 µM, 50 mL, obtained from Tocris Bioscience, Ellisville, MO, USA) or the type 1 cannabinoid receptor (CB1R) antagonist AM-251 (1 µM, 50 mL, obtained from Tocris Bioscience, Ellisville, MO, USA) was applied to prevent the decrease of the GABA release from the PV- and CB1R-positive perisomatic inhibitory cells, respectively. The antagonist was applied first on its own, then together with carbachol. At the end of the pharmacological experiment, the washout period endured for at least 30 min. Ten-minute-long epochs were recorded continuously during the whole pharmacological experiment (control–carbachol–washout no. 1–mAChR antagonist–mAChR antagonist with carbachol–washout no. 2).

### 4.6. Data Analysis

Data were analyzed with the Neuroscan Edit4.5 program (Compumedics Neuroscan, Charlotte, NC, USA), and home written routines for MATLAB (The MathWorks, Natick, MA, USA, RRID:SCR_001622). The microelectrode covered all layers of the neocortex. Usually, channels 1–12 were in the supragranular, channels 13–15 in the granular and channels 16-23 were in the infragranular layers. Channel positions were determined according to the thickness of the neocortex of the given patient and corrected if necessary.

Detection of the SPA was performed on LFPg records after a Hamming window spatial smoothing and a band-pass filtering between 1 and 30 Hz (zero phase shift, 48 dB/octave). Note that SPA is the totality of ‘SPA events’ occurring in a given recording (on average 286 ± 214 SPA events/recording). SPA events were visible usually on 5 to 16 channels, and SPA events larger than two times the standard deviation of the basal activity were detected and included in the analysis. The largest amplitude LFPg peak of the events was chosen as time zero for averaging. The location and the recurrence frequency of the SPA events were determined in each file during the whole recording session. To examine significance in the LFPg amplitude change induced by the different pharmacological agents, we used both the averaged LFPg amplitude and the LFPg amplitude of all SPA events occurring in a given recording. The latter will be called ‘individual LFPg amplitude’. The channel showing the largest LFPg amplitude on the averaged LFPg amplitude was chosen for the analysis of the individual LFPg amplitudes. Multiple unit activity (MUA) was calculated from the LFPg using standard techniques [15]. LFPg and MUA were averaged from −300 to +300 ms from the peak of the events determined as described above. Baseline correction (−150 to −50 ms) was applied to averaged LFPg and MUA.

Data are presented as median (first to third quartiles). Changes compared to physiological solution (due to pharmacological agents) were given in percentage of the values in physiological solution (median (first to third quartiles)).

### 4.7. Histology

Immunostainings were performed to visualize the two types of perisomatic inhibitory cells: the PV- and the CB1R-positive interneurons, as well as to verify the laminar structure and the possible tumor infiltration or signs of dysgenesis in the neocortex [15]. Neocortical slices following electrophysiological recording were immediately fixed with a fixative containing 4% paraformaldehyde and 15% picric acid in 0.1 M phosphate buffer (PB). Sections measuring 60-µm thick were made from the slices with a Leica VT1000S vibratome. Following washing in 0.1 M PB, the sections were immersed in 30% saccharose for 1–2 days, then frozen three times over liquid nitrogen. Sections were processed for immunostaining against the neuronal cell body marker NeuN antibody (1:2000, EMD Millipore, Billerica, MA, USA, RRID:AB_2298772), and the astroglial cell marker glial fibrillary acidic protein antibody (GFAP, 1:2000, EMD Millipore, Billerica, MA, USA, RRID:AB_94844), and the perisomatic inhibitory cell markers PV (1:7000, Swant, Bellinzona, Switzerland, RRID:AB_10000343) and CB1R (1:800, Cayman Chemicals, Ann Arbor MI, USA, RRID:AB_10080036). CB1Rs antibody binds to the receptors located in both glutamatergic and GABAergic axon terminals. The CB1R antibody was a rabbit polyclonal, all the other antibodies were mouse monoclonal antibodies. Their specificity was tested by the manufacturer. Sections were transferred to 0.1 M Tris-buffered saline (TBS, pH: 7.4), then endogenous peroxidase was blocked by 1% H_2_O_2_ in TBS for 10 min. TBS was used for all the washes (3 × 10 min between each serum) and for the dilution of the antisera. Non-specific immunostaining was blocked by 2% normal goat serum and 2% normal horse serum for one hour. The primary antibodies were applied for two days at 4 °C. For visualization of the immunopositive elements biotinylated anti-rabbit (in case of CB1R) or anti-mouse (in case of NeuN, GFAP and PV) IgG (1:250, Vector, Burlingame, CA, USA) was applied as secondary antibody (2 h), followed by avidin-biotinylated horseradish peroxidase complex (ABC, 1:250, 1.5 h, Vector, Burlingame, CA, USA). The immunoperoxidase reaction was developed by 3,3′-diaminobenzidine tetrahydrochloride dissolved in Tris buffer (pH: 7.6), as a chromogen. Sections were osmicated (0.5% OsO_4_ in 0.1M PB, 20 min), dehydrated in ethanol and mounted in Durcupan (ACM, obtained from Merck, Kenilworth, NJ, USA).

Since CB1R has a different laminar distribution across different cortical areas [46], for the electron microscopy we chose only samples derived from the temporal lobe. After light microscopic examination, in tissue derived from both ResEpi and NoEpi patients, areas of interest (regions generating SPA and regions not generating SPA in the same slice) were reembedded and sectioned for electron microscopy with a Leica EM UC7 ultramicrotome (Leica Biosystems, Buffalo Grove, IL, USA, RRID:SCR_016694). Layer 2/3 of the neocortex was chosen for the electron microscopy, as this was usually the region which generated SPA. Two regions of interest were examined in each slice, one generating SPA and a neighboring region that did not show SPA from PV- as well as from CB1R-immunostained sections. Ultrathin sections were collected on Formvar-coated single slot grids, stained with lead citrate and examined with either a Hitachi 7100 (Hitachi, Tokyo, Japan) or a Jeol JEM-1011 (Jeol, Tokyo, Japan) electron microscope. Both electron microscopes are equipped with a Mega-View III digital camera and a Soft Imaging Solutions (Olympus, Tokyo, Japan) image analyser system. Photographs were taken of every pyramidal cell body as well as of every bouton terminating on these somata. The immunopositivity or -negativity of the axon terminal was determined in each case. The asymmetric or symmetric nature (presumably excitatory or inhibitory, respectively) was determined for each synapse. Only one ultrathin section was examined in each sample, to avoid double sampling of the same cell or the same axon terminal. The perimeter of each cell body and the length of each synaptic active zone was measured with the Image J program (National Institute of Health, Bethesda, MA, USA). The number of synapses/100 µm soma perimeter, the synaptic coverage (µm synaptic length/100 µm soma perimeter) was determined for every cell, and the average synaptic active zone length was also determined in every region of interest. Altogether, the perisomatic synaptic coverage of 123 and 157 pyramidal cells were examined from 3 PV- and 3 CB1R-stained slices from NoEpi and 3 PV- and 3 CB1R-stained slices from ResEpi samples, derived from four and six patients, respectively.

The number of the examined cell bodies varied between 7 and 18 among the samples, with an average of 11.7 ± 3.7 per region of interest. Interneuronal and glial somata were excluded from the analysis, based on their morphological properties [72]. Inhibitory cells usually possess a considerably lower nucleus/cytoplasm ratio, have invaginated nucleus and do not possess the thick apical dendrite typical to pyramidal cells. The cytoplasm and the nucleus of glial cells are considerably more electron dense than that of neurons. The number of synapses/100µm soma perimeter, the synaptic coverage of the pyramidal cells and the average length of the active zones were determined in each region of interest (PV/SPA+, PV/SPA−, CB1R/SPA+, CB1R/SPA−) per slice, then averaged across patients belonging to the same group (ResEpi or NoEpi).

### 4.8. Statistics

#### 4.8.1. Histology

We determined the statistical significances with the aid of the program Statistica 13 (Tibco Software Inc., Palo Alto, CA, USA, RRID:SCR_014213). The lengths of the synaptic active zones (total, PV+, PV−, CB1R+, CB1R−) followed normal distribution (verified with the Kolmogorov–Smirnov and Lilliefors test), and statistical significances were examined with the Student’s *t*-test. The synaptic coverages (µm synaptic active zone/100 µm soma perimeter) and the numbers of synapses/100 µm soma perimeter values failed the normality test, therefore statistical differences were assessed with Mann–Whitney U test or Kruskal–Wallis ANOVA for comparing two or multiple groups, respectively. In cases of significant differences obtained with Kruskal–Wallis ANOVA, the *p* values were corrected with the Bonferroni adjustment. In order to test for unequal proportions in contingency tables we used Chi-square test.

#### 4.8.2. Electrophysiology

The statistical significances were examined with the aid of the R software (R Core Team 2020, R Foundation for Statistical Computing, Vienna, Austria). None of the data sets followed normal distribution (verified with the Shapiro–Wilk test). One-way repeated measures ANOVA was done to reveal presumable differences between the effects of the applied pharmacological agents (frequency, LFPg- and MUA amplitude) within each patient group (NoEpi or ResEpi). Pairwise differences were examined by post-hoc multiple pairwise paired *t*-tests with a Bonferroni adjustment. Welch’s *t*-test was done to reveal presumable differences (in the frequency, LFPg- and MUA amplitude) between the NoEpi and the ResEpi group. The significance level was set to *p* = 0.05.

## Figures and Tables

**Table 1:** Patient data. NoEpi: non-epileptic patients, ResEpi: patients with pharmacoresistant epilepsy, M: male, F: female. Electrophysiological experiments (detection of SPA) and light microscopy were performed in all samples, additional pharmacology (pharm) and/or electron microscopy (EM) were made in the designated samples (see Experiment column). Distance from the tumor: close <3 cm, distant >3 cm.

Stage of Epilepsy	Gender	Age	Duration of Epilepsy	Histology/Diagnosis	Resected Cortical Region	Experiment	Seizure Onset Zone	Distance from Tumor	Anatomy of Obtained Tissue
NoEpi	M	81		glioblastoma multiforme grade IV	temporal	EM		close	infiltrated/normal
NoEpi	M	32		anaplastic astrocytoma grade III	temporal	pharm		close	infiltrated
NoEpi	F	63		lung carcinoma metastasis	occipital	pharm		close	normal
NoEpi	M	64		lung adenocarcinoma metastasis	frontal	pharm		close	compressed neocortex
NoEpi	M	67		diffuse large B cell lymphoma	frontal	pharm		close	normal
NoEpi	F	78		glioblastoma grade IV	temporal	EM, pharm		distant	normal
NoEpi	F	65		glioblastoma multiforme	temporal	EM, pharm		close	normal
NoEpi	F	52		glioblastoma grade IV	parietal			distant	infiltrated
NoEpi	F	36		anaplastic ependymoma grade III	parietal	pharm		distant	normal
NoEpi	F	55		glioblastoma multiforme	frontal	pharm		distant	normal
NoEpi	F	59		breast carcinoma metastasis	frontal			close	infiltrated
NoEpi	F	68		glioblastoma multiforme grade IV	temporal	EM		close	normal
NoEpi	F	67		glioblastoma multiforme grade IV	parietal			close	normal
ResEpi	M	39	35 years	hippocampal sclerosis	temporal	EM	no	-	normal
ResEpi	F	53	40 years	hippocampal sclerosis	temporal	EM	no	-	normal
ResEpi	M	35	34 years	focal cortical dysplasia + hippocampal sclerosis	temporal	EM	no	-	normal
ResEpi	M	32	23 years	focal cortical dysplasia IIb with balloon cells	temporal	EM	no	-	normal
ResEpi	F	41	9 years	hippocampal and cortical gliosis, microglia activation	temporal	EM	no	-	dysgenetic
ResEpi	F	34	3 years	cortical gliosis	temporal	pharm	yes	-	normal
ResEpi	M	37	19 years	cortical gliosis, microglia activation	temporal	pharm	no	-	normal
ResEpi	M	32	19 years	ganglioglioma grade I	temporal		yes	close	normal
ResEpi	M	30	6 years	hippocampal sclerosis, reactive astrocytosis, microglia activation	temporal	pharm	no	-	normal
ResEpi	F	28	27 years	diffuse glioneural tumor grade I	temporal	EM, pharm	no	distant	normal
ResEpi	F	48	10 months	diffuse astrocytoma grade II	temporal	pharm	yes	close	normal
ResEpi	M	45	43 years	stroke induced lesion	frontal	pharm	yes	distant	dysgenetic

**Table 2:** Synaptic coverage of layer two pyramidal cells in the human neocortex, in NoEpi and ResEpi samples. Synaptic coverage = µm synaptic active zone/100 µm soma perimeter. Median (first to third quartiles) are provided. No significant differences were found between NoEpi and ResEpi, between SPA and No SPA, neither in the overall, or the PV+, CB1R+ synaptic coverage. Significant differences were found in the average length of PV+ and CB1R– synaptic active zones, as well as in the number of synapses/100 µm soma perimeter of synapses located in regions generating SPA, between NoEpi and ResEpi. Statistical differences in the length of the synaptic active zones were determined by Student’s *t*-test, whereas differences in the synaptic coverages and the numbers of synapses per 100 µm soma perimeter were assessed by Mann–Whitney U test (to compare two groups, such as ResEpi with NoEpi, or SPA with No SPA), and Kruskal–Wallis ANOVA to reveal statistical differences between four groups—i.e., (1) ResEpi SPA, (2) ResEpi No SPA, (3) NoEpi SPA, (4) NoEpi No SPA, with the Bonferroni adjustment. (** *p* < 0.01, * *p* < 0.05).

	NoEpi			ResEpi		
	Total	SPA	No SPA	Total	SPA	No SPA
Overall synaptic coverage	1.07 [0.62–1.50] *n* = 123 cells	1.07 [0.80–1.46] *n* = 57 cells	1.07 [0.55–1.52] *n* = 66 cells	1.12 [0.64–1.67] *n* = 156 cells	1.03 [0.56–1.51] *n* = 81 cells	1.23 [0.69–1.70] *n* = 75 cells
PV+ synaptic coverage	1.07 [0.58–1.52] *n* = 62 cells	0.52 [0.36–0.75] *n* = 26 cells	0.52 [0.00–0.90] *n* = 36 cells	1.18 [0.57–1.70] *n* = 78 cells	0.34 [0.00–0.68] *n* = 42 cells	0.35 [0.00–0.67] *n* = 36 cells
% of PV+ synaptic coverage	46.75 [0.00–58.41]	45.74 [0.00–52.86]	52.48 [0.00–75.41]	30.18 [0.00–54.44]	27.24 [0.00–53.68]	32.72 [0.00–58.29]
CB1R+ synaptic coverage	1.04 [0.68–1.46] *n* = 61 cells	0.42 [0.00–0.63] *n* = 31 cells	0.37 [0.00–0.68] *n* = 30 cells	1.05 [0.65–1.62] *n* = 78 cells	0.38 [0.00–0.58] *n* = 39 cells	0.62 [0.30–0.80] *n* = 39 cells
% of CB1R+ synaptic coverage	42.82 [0.00–57.28]	48.35 [0.00–59.98]	34.75 [0.00–51.16]	44.66 [22.23–62.72]	44.18 [0.00–63.62]	47.76 [31.33–58.73]
Average length of synaptic active zones (µm)	0.229 [0.201–0.260] *n* = 325 boutons	0.225 [0.198–0.255] *n* = 129 boutons	0.230 [0.201–0.267] *n* = 196 boutons	0.244 [0.211–0.270] *n* = 333 boutons	0.249 [0.213–0.274] *n* = 188 boutons	0.237 [0.207–0.266] *n* = 145 boutons
Average length of synaptic active zones of PV+ terminals	0.228 [0.200–0.256] *n* = 60 boutons	0.231 [0.203–0.253] ** n = 25 boutons	0.228 [0.196–0.263] *n* = 35 boutons	0.255 [0.228–0.275] *n* = 65 boutons	0.260 [0.243–0.276] ** n = 36 boutons	0.244 [0.217–0.275] *n* = 29 boutons
Average length of synaptic active zones of PV− terminals	0.228 [0.194–0.257] *n* = 77 boutons	0.234 [0.201–0.258] *n* = 40 boutons	0.212 [0.179–0.249] *n* = 37 boutons	0.241 [0.212–0.262] *n* = 129 boutons	0.240 [0.209–0.259] *n* = 73 boutons	0.242 [0.213–0.264] *n* = 56 boutons
Average length of synaptic active zones of CB1R+ terminals	0.229 [0.196–0.264] *n* = 77 boutons	0.216 [0.193–0.255] *n* = 26 boutons	0.241 [0.198–0.277] *n* = 51 boutons	0.226 [0.193–0.282] *n* = 61 boutons	0.237 [0.154–0.291] *n* = 34 boutons	0.226 [0.195–0.253] *n* = 27 boutons
Average length of synaptic active zones of CB1R− terminals	0.230 [0.208–0.263] *n* = 109 boutons	0.219 [0.199–0.245] * n = 36 boutons	0.241 [0.211–0.276] *n* = 73 boutons	0.248 [0.212–0.273] *n* = 78 boutons	0.254 [0.233–0.276] * n = 45 boutons	0.226 [0.190–0.259] *n* = 33 boutons
Average number of PV+ synapses/100 µm soma perimeter	2.19 [0.00–3.51] *n* = 62 cells	2.23 [1.36–3.51] ** n = 26 cells	2.17 [0.00–3.48] *n* = 36 cells	0.25 [0.00–2.06] *n* = 78 cells	0.23 [0.00–1.90] ** n = 42 cells	1.00 [0.00–2.14] *n* = 36 cells
Average of PV− synapses/100 µm soma perimeter	2.49 [1.71–4.09] *n* = 62 cells	2.87 [2.12–4.41] * n = 26 cells	2.17 [0.00–2.76] *n* = 36 cells	2.13 [0.30–3.81] *n* = 78 cells	2.15 [0.24–3.81] * n = 42 cells	2.09 [1.21–3.56] *n* = 36 cells
Average of CB1R+ synapses/100 µm soma perimeter	2.07 [0.00–2.80] *n* = 61 cells	2.21 [0.00–3.04] *n* = 31 cells	1.87 [0.00–2.47] *n* = 30 cells	2.02 [0.00–3.20] *n* = 77 cells	1.87 [0.00–2.63] *n* = 38 cells	2.09 [1.41–3.64] *n* = 39 cells
Average number of CB1R− synapses/100 µm soma perimeter	2.62 [2.03–4.23] *n* = 61 cells	2.47 [1.89–3.95] *n* = 31 cells	3.62 [2.07–4.42] *n* = 30 cells	2.65 [1.80–4.53] *n* = 77 cells	2.55 [1.31–4.95] *n* = 38 cells	2.82 [1.94–4.53] *n* = 39 cells

**Table 3:** Recurrence frequency of SPAs. Black asterisk: significant difference between physiological solution and pharmacological agent (one-way repeated measures ANOVA, *p* < 0.05). Red asterisk: significant difference between NoEpi and ResEpi (Welch’s *t*-test, *p* < 0.01). Data are presented as median (first to third quartiles). Changes compared to baseline (physiological solution) were given in percentage of the baseline.

Frequency (Hz)	NoEpi					ResEpi			
		Physiological Solution	Pharmacological Agent	Changes Compared to Physiological Solution (%)			Physiological Solution	Pharmacological Agent	Changes Compared to Physiological Solution (%)
Carbachol	*n* = 12	1.37 [0.74–1.76]	1.24 [0.59–1.57]	80 [72–95]	*	*n* = 8	0.88 [0.67–1.84]	0.10 [0.02–0.37] *	9 [3–27]
AF-DX 116	*n* = 3	0.98 [0.85–0.99]	0.97 [0.77–1.02]	99 [88–102]		*n* = 4	1.28 [0.99–1.56]	1.33 [1.15–1.48]	102 [99–107]
AF-DX 116 + Carbachol	*n* = 6	0.99 [0.78–1.45]	0.82 [0.72–1.19]	78 [75–87]		*n* = 4	1.28 [0.99–1.56]	0.90 [0.87–1.07]	83 [71–96]
AM-251	*n* = 3	1.02 [0.61–1.21]	1.07 [0.63–1.18]	92 [91–99]		*n* = 5	0.94 [0.81–1.71]	0.97 [0.93–1.49]	96 [94–115]
AM-251 + Carbachol	*n* = 5	1.40 [1.23–1.51]	1.47 [1.03–1.50]	88 [84–97]		*n* = 5	0.94 [0.81–1.71]	0.83 [0.02–0.94]	88 [2–93]

**Table 4:** LFPg amplitude of SPA. Black asterisk: significant difference between physiological solution and pharmacological agent in case of averaged LFPg amplitude (one-way repeated measures ANOVA, *p* < 0.05). Grey asterisk: significant difference between physiological solution and pharmacological agent in case of LFPg amplitude of individual SPA events (one-way repeated measures ANOVA, *p* < 0.01). Dark blue asterisk: significant difference between carbachol and pharmacological agent in case of averaged LFPg amplitude (one-way repeated measures ANOVA, *p* < 0.05). Light blue asterisk: significant difference between carbachol and pharmacological agent in case of LFPg amplitude of individual SPA events (one-way repeated measures ANOVA, *p* < 0.01). Red asterisk: significant difference between NoEpi and ResEpi in case of averaged LFPg amplitude (Welch’s *t*-test, *p* < 0.05). Light red asterisk: significant difference between NoEpi and ResEpi in case of LFPg amplitude of individual SPA events (Welch’s *t*-test, *p* < 0.01). Data are presented as median (first to third quartiles). Changes compared to baseline (physiological solution) were given in percentage of the baseline.

LFPg Amplitude (µV)	NoEpi					ResEpi			
		Physiological Solution	Pharmacological Agent	Changes Compared to Physiological Solution (%)			Physiological Solution	Pharmacological Agent	Changes Compared to Physiological Solution (%)
Carbachol	*n* = 12	36.30 [25.01–46.44]	23.42 [20.00–31.01] **	68 [63–77]		*n* = 8	66.01 [36.46–106.49]	18.10 [12.20–31.21] **	37 [25–45]
AF-DX 116	*n* = 3	38.67 [27.55–38.75]	34.88 [25.06–41.60] *	93 [91–109]	* *	*n* = 4	79.43 [60.50–95.75]	79.70 [59.51–96.74] *	99 [99–100]
AF-DX 116 + Carbachol	*n* = 6	38.75 [38.08–62.72]	34.28 [28.52–39.61] **	92 [75–96]	* *	*n* = 4	79.43 [60.50–95.75]	63.87 [53.55–76.51] ***	87 [83–91]
AM-251	*n* = 3	35.48 [29.46–41.67]	29.71 [28.06–41.98] *	113 [98–113]	*	*n* = 5	55.66 [46.00–89.09]	63.13 [46.15–88.51] *	100 [99–100]
AM-251 + Carbachol	*n* = 5	39.80 [27.18–46.92]	22.81 [22.34–34.12] **	68 [64–95]	*	*n* = 5	55.66 [46.00–89.09]	22.78 [11.18–33.91] ***	38 [34–50]

**Table 5:** MUA amplitude. Red asterisk: significant difference between NoEpi and ResEpi (Welch’s *t*-test, *p* < 0.05). Data are presented as median (first to third quartiles). Changes compared to baseline (physiological solution) were given in percentage of the baseline.

MUA Amplitude (µV)	NoEpi					ResEpi			
		Physiological Solution	Pharmacological Agent	Changes Compared to Physiological Solution (%)			Physiological Solution	Pharmacological Agent	Changes Compared to Physiological Solution (%)
Carbachol	*n* = 12	0.67 [0.56–1.10]	0.67 [0.46–0.85]	81 [57–107]		*n* = 8	3.12 [1.82–5.36]	1.21 [0.41–3.03]	43 [22–85]
AF-DX 116	*n* = 3	0.98 [0.83–1.10]	0.94 [0.81–1.22]	97 [97–110]	*	*n* = 4	3.89 [3.73–4.41]	4.89 [4.20–5.57]	113 [102–127]
AF-DX 116 + Carbachol	*n* = 6	1.10 [0.76–1.41]	0.75 [0.69–0.95]	88 [80–97]	*	*n* = 4	3.89 [3.73–4.41]	4.93 [4.21–5.31]	111 [86–135]
AM-251	*n* = 3	0.87 [0.66–1.04]	1.24 [0.90–1.99]	124 [114–219]		*n* = 5	4.07 [2.84–5.20]	3.73 [2.89–4.13]	92 [88–102]
AM-251 + Carbachol	*n* = 5	0.87 [0.73–1.20]	1.32 [1.27–1.49]	139 [122–155]		*n* = 5	4.07 [2.84–5.20]	2.50 [1.50–4.04]	65 [64–88]

## Data Availability

The data supporting the findings of this study are available on request from the corresponding author.
